# Blood Pressure Profile and Hypertensive Organ Damage in COPD Patients and Matched Controls. The RETAPOC Study

**DOI:** 10.1371/journal.pone.0157932

**Published:** 2016-06-30

**Authors:** Rafael Golpe, Alfonso Mateos-Colino, Ana Testa-Fernández, Marta Pena-Seijo, Manuel Rodríguez-Enríquez, Carlos González-Juanatey, Francisco J. Martín-Vázquez, Antonio Pose-Reino, Nuria Domínguez-Pin, Nuria Garnacho-Gayarre, Luis A. Pérez-de-Llano

**Affiliations:** 1 Respiratory Medicine Service, University Hospital Lucus Augusti, Lugo, Spain; 2 Internal Medicine Service, University Hospital Lucus Augusti, Lugo, Spain; 3 Cardiology Service, University Hospital Lucus Augusti, Lugo, Spain; 4 Internal Medicine Service, Complexo Hospitalario Universitario de Santiago, Santiago de Compostela, Spain; 5 Ophthalmology Service, University Hospital Lucus Augusti, Lugo, Spain; 6 EOXI Lugo, Cervo e Monforte de Lemos, Galician Health Service, Lugo, Spain; 7 Laboratory Service, University Hospital Lucus Augusti, Calle Dr. Ulises Romero n°1, 27003, Lugo, Spain; Rouen University Hospital, FRANCE

## Abstract

**Background:**

Several studies suggest that there is a pathogenic link between chronic obstructive pulmonary disease (COPD) and cardiovascular diseases. On the other hand, increased sympathetic tone has been described in several respiratory diseases. Our objective was to determine whether hypertension mediated by sympathetic overactivity is a mechanism that explains the association between COPD and cardiovascular diseases.

**Methods:**

Prospective nested case-control observational study; 67 COPD patients were matched 1:1 by sex and age to controls with smoking history. 24 hour-blood pressure monitoring, urinary catecholamines and their metabolites measurement, echocardiography, carotid ultrasound examination, nocturnal oximetry and retinography were performed.

**Findings:**

classic cardiovascular risk factors and comorbidities were similarly distributed between cases and controls. No significant differences for blood pressure variables (difference for mean systolic blood pressure: -0·13 mmHg; 95% CI: -4·48,4·20; p = 0·94; similar results for all blood presssure variables) or catecholamines values were found between both groups. There was a tendency for lower left ventricle ejection fraction in the COPD cases, that approached statistical significance (64·8 ± 7·4 vs 67·1 ± 6·2, p = 0·05). There were no differences in the retinal arteriovenous ratio, the carotid intima-media thickness, or the number of carotid plaques, between cases and controls. Fibrinogen values were higher in the COPD group (378·4 ± 69·6 vs 352·2 ± 45·6 mg/dL, p = 0·01) and mean nocturnal oxygen saturation values were lower for COPD patients (89·0 ± 4·07 vs 92·3 ± 2·2%, p < 0·0001).

**Interpretation:**

Hypertension induced by sympathetic overactivity does not seem to be a mechanism that could explain the association between COPD and cardiovascular disease.

## Introduction

The World Health Organization estimates that chronic obstructive pulmonary disease (COPD) will become the 3^rd^ leading cause of death worldwide in 2030 [1]. Observational data indicate that COPD is associated with a higher risk of cardiovascular diseases (CVD) [2,3]. Some authors hypothesized that there is a pathogenic link between COPD and CVD. Increased levels of inflammatory cytokines are found in COPD and this persistent, low-grade systemic inflammation might contribute to the development of atherosclerotic vascular changes [4]. However, shared risk factors (age, sex, smoking, low physical activity), might also explain the association between COPD and CVD. In fact, some studies did not find a clear connection between CVD and COPD when classical cardiovascular risk factors were taken into account [5,6]. Underuse of cardiac medications, especially beta-blockers, in COPD might explain the higher risk of cardiovascular events in this population [6]. Therefore, there is a need for more research on this topic.

Atherosclerosis is associated with an increased risk of stroke, coronary artery disease, peripheral arterial disease and mortality [7], but other additional factors beyond inflammation-mediated atherosclerosis might explain cardiovascular mortality in COPD. Hypertension is a major cardiovascular risk factor, and it is a frequent finding in other respiratory diseases, like obstructive sleep apnea (OSA) [8]. Hypertension in these cases could be related to increased sympathetic tone [9]. However, there is conflicting information about the relationship between hypertension and COPD. Two studies found an increased prevalence of hypertension among patients with COPD [10,11], while other studies that focused on COPD prevalence among patients with hypertension failed to find an association [3]. Anderson et al found higher systolic blood pressure (BP) in 93 COPD patients compared with 34 control subjects. However, the study groups were not well matched [12]. Few studies have evaluated the pattern of BP (e.g.: percentage of dippers vs. nondippers) in COPD patients [13], and we are not aware of any study that evaluates the type of organ damage due to hypertension in matched cohorts of COPD patients and controls.

Our main objective was to assess if BP in patients with COPD has a different profile than in matched controls with a history of smoking and if the differences might be related to sympathetic tone. That would suggest a pathogenic link between COPD and CVD. The secondary objectives of our study (Hypertension Repercussion in COPD, abbreviated to RETAPOC in Spanish), were to evaluate if hypertensive organ damage was different in COPD cases.

## Methods

### Study design and subjects

**Fig 1:**
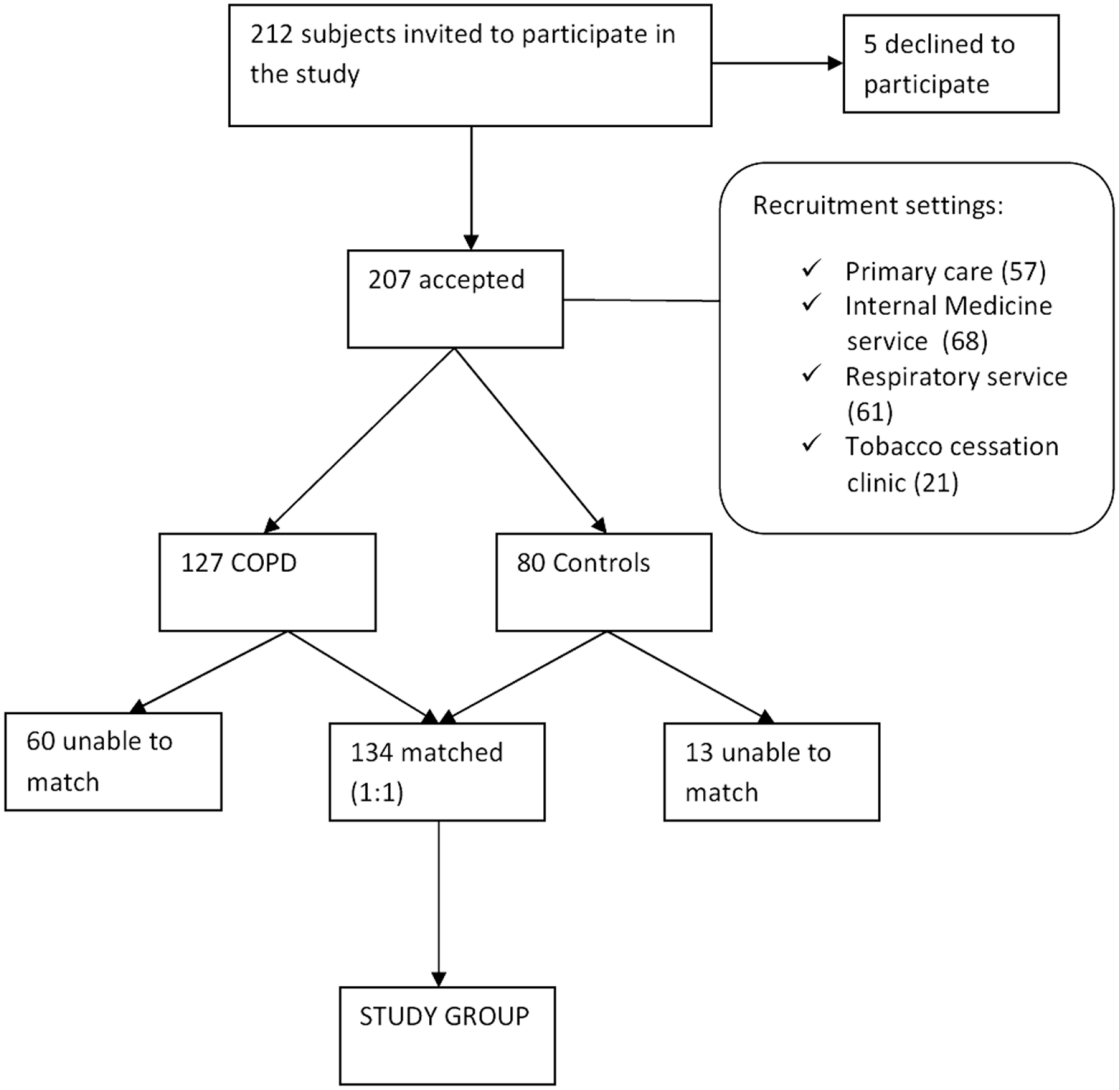
Flow diagram of the study.

This is a prospective, 1:1 matched, case-control observational, non-interventional study. The study included men and women with a history of smoking. Subjects were invited to participate when they attended either their primary care clinic (usually, for a standard health check-up), or the internal medicine clinic, the respiratory clinic or the tobacco cessation program of our hospital (Fig 1). The recruitment period was September 21, 2011 to October 25, 2014.

Inclusion criteria were: smoking history with a pack-years index > 10, and age > 40 years. Exclusion criteria were: uncontrolled neoplasm, diagnosis of other inflammatory diseases (e.g.: collagen vascular diseases), known respiratory diseases other than COPD, morbid obesity, pregnancy, spirometry that was neither normal nor obstructive, and refusal or incapacity to give informed consent. A COPD case was defined by a FEV_1_/FVC ratio < 0·70 in post-bronchodilator spirometry. Each COPD subject was matched 1-to-1 for sex and age with one control subject with normal spirometry. Age was matched via nearest neighbor matching with a maximum allowable difference of 5 years between two participants. COPD subjects for whom no control case was found within the allowed limits were left unmatched and excluded from analysis.

Medical information was obtained through a questionnaire, which included demographic background, socioeconomic status, life habits and medical history. Dyspnea was evaluated with the modified medical research council (mMRC) scale. Comorbidity was assessed by means of the non-age adjusted Charlson index [14]. To compare comorbidities between both study groups, we used a modification of the index, excluding COPD from the score.

The study was performed in accordance with the 1964 Declaration of Helsinki and its later amendments and it was approved by our ethical committee (Comité Autonómico de Ética de la Investigación de Galicia, registry number: 2010/228). Informed written consent was obtained from all the subjects.

### Spirometry

Spirometry was performed according to the American Thoracic Society/European Respiratory Society consensus guidelines [15], and using the European Respiratory Society predicted values [16].

### Blood pressure monitoring

Twenty-four hour BP monitoring was performed with Spacelabs 90207 (Spacelabs Inc, Richmond, WA, USA). The non-dominant arm was used for measurement. Patients were instructed to maintain their usual activities, avoiding strenuous exercise. The monitors were programmed to obtain BP readings every 20 minutes between 8:00 h and 22:00 h and every 30 minutes between 22:00 h and 8:00 h. Daytime ambulatory BP was defined as the period between 8:00 and 22:00 h and nighttime as the period between 22:00 and 8:00 h. We classified the patients by nocturnal systolic BP fall as follows: extreme-dippers if the fall was ≥ 20%, dippers if the fall was ≥ 10% but < 20%, non-dippers if it was ≥ 0% but < 10% and risers if it was < 0% [17].

### Laboratory data

Venous blood was obtained in the morning and after an overnight fast. In order to reduce possible influence of daytime activity on the excretion of catecholamines, urine was collected for 12-hours, starting in the evening, after discarding the first voided sample [18]. Urinary epinephrine, norepinephrine, metanephrine and normetanephrine were measured by high resolution liquid chromatography (Bio-Rad laboratories, Hercules, CA).

### Echocardiography

Echocardiographies were performed by a cardiologist blinded to the clinical information, using a Philips IE33 (Philips Healthcare, Andover, MA) ultrasound imaging system with a S4 transducer. M-mode, two-dimensional, Doppler (continuous and pulsed wave), and color Doppler were performed. Left ventricle (LV) systolic function was assessed in most cases by Teichholz method and Simpsons’s biplane method. In some cases, it was assessed by visual estimation. LV systolic depression was defined as left LV ejection fraction < 50%. Right ventricle (RV) dilatation was defined as end-diastolic diameter > 30 mm or right-to-left ventricular end-diastolic diameter ratio ≥ 1 in apical 4-chamber view. Systolic pulmonary artery pressure (SPAP) was derived from the right atrioventricular pressure gradient using peak velocity of tricuspid flow regurgitation with continuous wave Doppler in the apical four chamber view and the modified Bernoulli equation [19]. Right atrial pressure was considered equal to 5 or 10 mmHg according to whether or not the inferior vena cava collapsed during inspiration, respectively [20]. Because no patient had RV outflow obstruction, SPAP was considered equivalent to RV systolic pressure. RV systolic function was assessed using tricuspid annular plane systolic excursion (TAPSE) and lateral RV annular peak systolic velocity by pulse wave tissue Doppler. A TAPSE < 15 mm was considered as indicative of RV dysfunction.

### Retinography

Twenty to 30 min. after pupil dilation with 2 drops of tropicamide and phenylephrine, an eye fundus photograph centered on the papilla, and covering an angle of 50°, was taken with a digital fundus camera equipped with a 540 nm filter and connected to an Imagenet I-base system (Topcon Instruments, Paramus, NJ, USA). A previously validated computer system was used to measure the calibers of retinal blood vessel, as described elsewhere [21]. The program calculates the average of all the calibers measured at arterial intersections, the average of all the calibers measured at venous intersections, and the ratio between these 2 averages (the arteriovenous ratio, AVR) [21].

### Carotid ultrasound examination

We used high-resolution B-mode ultrasound, Philips IE33 (Philips Healthcare, Andover, MA, USA), with a 10-MHz linear transducer. Carotid intima-media thickness (IMT) was measured at the far wall of the right and left common carotid arteries, 10 mm from the carotid bifurcation, over the proximal 10 mm long segment. IMT was determined using an automatic software C-IMT in Qlab system. The final IMT was the largest average IMT (left or right). The plaque criteria in the accessible extracranial carotid tree were defined according to Mannheim consensus [22]. The examinator was blinded to clinical information. The pulsatility and resistance indices were determined using automatic calculation software from the Doppler spectrum in the left and right common carotid arteries: pulsatility index = (systolic velocity—diastolic velocity) / mean velocity. Resistance index = (systolic velocity—diastolic velocity) / systolic velocity.

### Nocturnal oximetry

Nocturnal oxygen saturation (SpO_2_) was measured with a finger pulse oximeter (Pulsox 3i; Minolta, Ramsey NJ). The mean nighttime SpO_2_ (MNSpO_2_), percentage of recording time with SpO_2_ <90% (CT90) and the number of dips in SpO_2_ ≥4% per hour of recording time (DI4), were calculated using computer software (Pulsox SaO_2_ analysis software DS-3; Minolta, Ramsey NJ).

### Statistical analysis

Normal distribution of data was assessed using the D’Agostino-Pearson test. Between-groups comparisons for continuous variables were made using the *T*-Student or the Mann-Whitney tests, as appropriate. Chi-square test was used for categorical variables. Correlation between variables was assessed using Spearman’s rank correlation coefficient. A 2-tailed value of p < 0·05 was considered significant. Sample size was estimated as follows: Aidar et al found a difference of 15 mmHg in mean 24 h-systolic BP between COPD and controls with SD of 11·53 and 10·18 [13]. A sample size of 30 subjects would be required to detect a difference with these values. The difference was smaller (8 mmHg) in another study, and a sample size of 98 (with similar values for SD) would be required using these figures [12]. We took a prudent approach regarding sample size, and decided to include at least 120 study subjects. In order to explore the possibility of systematic bias, we (1^st^) compared the main characteristics of those case matched with controls and those lost to matching process and (2^nd^) performed multiple regression analysis by means of a stepwise forward selection using systolic BP as the dependent variable. We ruled out multicollinearity checking the variance inflation factor values and we used an automatic weighted regression procedure to take into account heteroscedasticity [23].

## Results

Five subjects refused to participate in the study. Two hundred and seven patients (127 COPD patients and 80 subjects with normal lung function) accepted to participate. Of this number, 67 COPD cases could be matched with 67 controls, and were included in the analysis (Fig 1). FEV_1_ was ≥ 80% for 11 COPD cases, 79–50% for 30, 40–30% for 22, and < 30% for 4 cases. Table 1 shows the characteristics of both groups. Classic cardiovascular risk factors and socioeconomic conditions (marital status, education level, employment status) were similarly distributed between both groups. The percentages of patients who performed daily exercise was also similar in both groups, but exercise was self-reported and, therefore, bias in this respect cannot be totally ruled out. The weight of comorbidities was also comparable, if COPD was not taken into account. There were more active smokers in the control group, and the cumulative smoking exposure was higher in the COPD group. Dyspnea was worse in the cases group, and the body mass index was lower in these subjects. Differences between COPD patients that could be matched to controls and those lost to matching process are shown in the S1 File (Table A in S1 File). As expected, patients that could not be matched were older, and all of them were males.

**Table 1:** Main characteristics of the cohort.

Variable	Cases (n = 67) COPD	Controls (n = 67) Smokers, non-COPD	P
**Age, yr**	59·4 ± 5·9	58·2 ± 6·9	0·28
**Males, n, %**	52 (77·6%)	52 (77·6%)	-
**Charlson index**	2·0± 1·2	0·7 ± 0·9	< 0·0001
Modified Charlson index*	0·9 ± 1·1	0·7 ± 0·9	0·14
**Active smokers, n, %**	34 (50·7%)	47 (70·1%)	0·001
**Pack-years**	52·7 ± 24·4	43·7 ± 26·9	0·01
**Alcohol intake > 80 gr/d, n, %**	10 (14·9%)	9 (13·4%)	1·00
**Treatment for hypertension, n, %**	29 (43·2%)	25 (37·3%)	0·59
**Treatment for dyslipidemia, n, %**	16 (23·8%)	24 (35·8%)	0·18
**Treatment for diabetes, n, %**	9 (13·4%)	13 (19·4%)	0·48
**Anticoagulation, n, %**	3 (4·4%)	0	0·24
**Antiaggregation, n, %**	18 (26·8%)	16 (23·9%)	0·84
**Chronic bronchitis, n, %**	44 (65·6%)	23 (34·3%)	0·0005
**Dyspnea mMRC**			< 0·0001
**mMRC class 0, n, %**	10 (14·9%)	38 (56·7%)	
**mMRC class 1, n, %**	29 (43·2%)	23 (34·3%)	
**mMRC class 2, n, %**	24 (35·8%)	6 (8·9%)	
**mMRC class 3, n, %**	4 (5·9%)	0	
**mMRC class 4, n, %**	0	0	
**Snoring n,%**	33 (49·2%)	30 (44·7%)	0·79
**Epworth value**	3·2 ± 2·1	3·7 ± 2·7	0·61
**BMI, Kg/m**^**2**^	27·7 ± 4·5	29·4 ± 4·5	0·03
**Abdominal perimeter, cm**	100·4 ± 12·2	100·3 ± 12·3	0·96
**FEV**_**1**_**%**	58·4 ± 20·7	96·6 ±15·8	< 0·0001
**FVC %**	82·4 ± 16·8	94·5 ± 13·6	< 0·0001
**FEV1/FVC %**	51·3 ± 12·3	77·1 ± 4·3	< 0·001

mMRC: modified medical research council; BMI: body mass index.

*In the modified Charlson index, COPD does not score.

BP monitoring results are summarized in Table 2. All the BP variables were similar between groups.

**Table 2:** Results of the ambulatory blood pressure monitoring.

Variable	Cases (n = 67) COPD	Controls (n = 67) Smokers, non-COPD	Difference (95% CI)	P
**24 h mean SBP, mmHg**	124·8 ± 12·6	124·7 ± 12·6	‒ 0·13 (‒ 4·48 to 4·20)	0·94
**24 h maximal SBP, mmHg**	155·3 ± 17·2	154·8 ± 18·3	‒ 0·45 (‒ 6·55 to 5·65)	0·88
**24 h mean DBP, mmHg**	76·1 ± 8·0	76·3 ± 6·6	0·19 (‒ 2·33 to 2·73)	0·87
**24 h maximal DBP, mmHg**	99·5 ± 10·4	99·2 ± 9·9	‒ 0·23 (‒ 3·73 to 3·26)	0·89
**Daytime mean SBP, mmHg**	127·8 ±12·6	128·9 ± 13·0	1·04 (‒ 3·34 to 5·43)	0·64
**Daytime maximal SBP, mmHg**	154·4 ± 16·0	154·3 ± 18·4	‒ 0·34 (‒ 5·96 to 5·88)	0·99
**Daytime mean DBP, mmHg**	79·3 ± 8·2	80·1 ± 7·1	0·71 (‒ 1·92 to 3·36)	0·59
**Daytime maximal DBP, mmHg**	99·0 ± 10·3	98·9 ± 10·1	‒ 0·07 (‒ 3·58 to 3·44)	0·96
**Nighttime mean SBP, mmHg**	119·7 ± 15·2	117·9 ± 13·4	‒ 1·79 (‒ 6·72 to 3·14)	0·47
**Nighttime maximal SBP, mmHg**	141·4 ± 18·2	138·4 ± 17·4	‒ 3·02 (‒ 9·14 to 3·10)	0·33
**Nighttime mean DBP, mmHg**	70·9 ± 9·2	70·2 ± 7·1	‒ 0·63 (‒ 3·46 to 2·18)	0·65
**Nighttime maximal DBP, mmHg**	87·6 ± 11·0	86·6 ± 10·4	‒ 1·05 (‒ 4·73 to 2·63)	0·57
**Non dipper, n, %**	32 (47·7%)	38 (56·7%)		0·15
**Dipper, n, %**	23 (34·3%)	22(32·8%)		
**Extreme dipper, n,%**	0	2 (2·9%)		
**Riser, n,%**	12 (17·9%)	5 (7·4%)		

CI: confidence interval; SBP: systolic blood pressure; DBP: diastolic blood pressure.

We repeated the analysis including only patients without previous treatment for hypertension, with similar results (Table B in S1 File). Forty eight patients (71.6%) in the COPD group and 42 (62.7%) subjects in the control group had hypertension (p = 0.36). The prevalence of occult hypertension, in subjects who did not receive previously antihypertensive treatment was 50% for cases and 40·4% for controls (p = 0·52) (Table C in S1 File). Urinary catecholamines and metabolites values were similar between groups (Table 3).

**Table 3:** Catecholamines and metabolites analysis results.

Variable	Cases (n = 67) COPD	Controls (n = 67) Smokers, non-COPD	P
**Epinephrine (μg/d)**	2·33 ± 2·11	2·15 ± 1·55	0·69
**Norepinephrine (μg/d)**	21·9 ± 11·5	23·6± 16·6	0·94
**Metanephrine (μg/d)**	27·9 ± 20·9	35·2 ± 34·9	0·72
**Normetanephrine (μg/d)**	63·9 ± 58·7	69·3 ± 78·0	0·55

Table 4 shows the blood analysis results. The only differences between groups were higher levels of fibrinogen in the COPD group and lower values of HDL-cholesterol in the control group.

**Table 4:** Blood analysis results.

Variable	Cases (n = 67) COPD	Controls (n = 67) Smokers, non-COPD	P
**Hemoglobin, gr/dL**	14·7 ± 1·4	14·5 ± 1·2	0·43
**Leukocyte count, 1000/mm**^**3**^	7·6 ± 2·0	7·3 ± 1·9	0·32
**Neutrophils %**	59·7 ± 7·6	58·8 ± 8·9	0·57
**ESR, mm/h**	10·7 ± 9·2	8·5 ± 7·3	0·13
**Fibrinogen, mg/dL**	378·4 ± 69·6	352·2 ± 45·6	0·01
**D dimer, ng/mL**	169·9 ± 167·4	122·3 ± 124·3	0·08
**Glucose, mg/dL**	110·9 ± 29·3	117·7 ± 40·9	0·27
**Urea, mg/dL**	42·8 ± 16·2	39·7 ±10·9	0·21
**Creatinine, mg/dL**	0·8 ± 0·2	0·8 ± 0·1	0·27
**Albumin, g/dL**	4·4 ± 0·2	4·3 ± 0·2	0·91
**Cholesterol, mg/dL**	199·5 ± 45·4	208·5 ± 40·3	0·23
**HDL-cholesterol, mg/dL**	54·6 ± 15·5	48·0 ± 12·4	0·01
**Triglycerides, mg/dL**	129·7 ± 59·8	158·2 ± 105·8	0·06
**CRP, mg/L**	5·5 ± 11·2	4·1 ± 5·0	0·40

ESR: erythrocyte sedimentation rate; CRP: C-reactive protein.

Table 5 summarizes the echocardiography findings. Left atrial size was slightly higher in the control group. There was a tendency towards lower LV ejection fraction in the COPD group, that was near the level of statistical significance. No other significant differences were found.

**Table 5:** Echocardiography results.

Variable	Cases (n = 67) COPD	Controls (n = 67) Smokers, non-COPD	P
**LV dilatation, n, %**	1 (1·5%)	0	1·00
**LV hypertrophy, n, %**	14 (20·9%)	13 (19·4%)	1·00
**LV hypokinesis, n, %**	6 (8·9%)	3 (4·4%)	0·49
**LV systolic dysfunction, n, %**	3 (4·5%)	1 (1·5%)	0·61
**LV relaxation pattern, n, %**			0·35
**Normal**	36 (53·7%)	39 (58·2%)	
**Abnormal**	29 (43·3%)	23 (34·3%)	
**Pseudonormal**	2 (2·9%)	5 (7·4%)	
**Restrictive**	0	0	
**RV dilatation, n, %**	4 (5·9%)	1 (1·5%)	0·36
**RV dysfunction, n, %**	0	0	-
**Valvular dysfunction, n, %**	3 (4·5%)	3 (4·5%)	0·67
**IVC dilatation, n, %**	0	0	-
**LVDD, mm**	45·6 ± 7·2	46·5 ± 4·9	0·42
**LVSD, mm**	28·1 ± 9·0	28·7 ± 4·9	0·65
**IVS, mm**	11·8± 11·1	10·2 ± 1·7	0·27
**LVPW, mm**	9·4 ± 2·2	9·5 ± 1·9	0·71
**LVSF, %**	35·9 ± 4·5	37·5 ± 5·0	0·07
**LVEF, %**	64·8 ± 7·4	67·1 ± 6·2	0·05
SPAP, mmHg*	27·4 ± 6·2	26·5 ± 3·4	0·46
**E/E’**	8·4 ± 2·1	8·4 ± 2·6	0·95
**LA, mm**	33·6 ± 6·7	36·2 ±4·1	0·01
**TAPSE, mm**	22·4 ± 2·9	23·0 ± 3·4	0·27

LV: left ventricle; RV: right ventricle; IVC: inferior vena cava; LVDD: left ventricle diastolic diameter; LVSD: left ventricle systolic diameter; IVS: interventricular septum (diastole); LVPW: left ventricle posterior wall; LVSF: left ventricle shortening fraction; LVEF: left ventricle ejection; SPAP: systolic pulmonary artery pressure; LA: left atrial size; TAPSE: tricuspid annular plane systolic excursion.

*Tricuspid regurgitation signal was adequate for SPAP measurement in 38 cases and 42 controls.

Table 6 displays the carotid ultrasound results. The IMT was higher in COPD patients, but the difference with the control group failed to achieve statistical significance. The number of carotid plaques was similar between groups.

**Table 6:** Carotid ultrasound results.

Variable	Cases (n = 67) COPD	Controls (n = 67) Smokers, non-COPD	P
**IMT, mm**	0·81 ± 0·23	0·75 ± 0·19	0·08
**PI**	1·77 ± 0·49	1·70 ± 0·50	0·40
**RI**	0·72 ± 0·11	0·71 ± 0·06	0·69
**Carotid plaques, n, %**	37 (55·2%)	31 (46·2%)	0·38

IMT: intima media thickness; PI: pulsatility index; RI: resistance index.

Only 72 nocturnal oximetries (40 in cases and 32 in controls) were performed, because of technical problems with the equipment. In order to rule out significant bias, we compared the characteristics of those subjects in which oximetry was performed with those in which it was not possible. No differences were found, except for a slightly higher FVC in COPD cases in which oximetry was performed (Table D in S1 File). Table 7 shows the results of the nocturnal oximetry. MnSpO_2_ was lower and CT90 was higher in the cases group. DI4 and the percentage of subjects with a saw-tooth saturation pattern were similar between groups.

**Table 7:** Results of the nocturnal oximetry.

Variable	Cases (n = 40) COPD	Controls (n = 32) Smokers, non-COPD	P
**MNSpO**_**2**_**, %**	89·0 ± 4·07	92·3 ± 2·2	< 0·0001
**CT90, %**	48·6 ± 32·7	17·8 ± 21·5	< 0·0001
**DI4, n°/h**	14·1 ± 13·8	14·1 ± 13·6	0·64
**Sawtooth pattern, n, %**	15 (37·5%)	14 (43·7%)	0·85

MNSpO_2_: mean nighttime oxygen saturation; CT90: percentage of recording time with SpO_2_ <90%; DI4: number of dips in SpO_2_ ≥4% per hour of recording time.

We searched for correlations between catecholamines values and results of 24-h BP monitoring. There was a significant, although weak, correlation between norepinephrine and systolic BP values. No other correlations were found (Table E in S1 File).

We could not obtain quality measurement of the retinal AVR in 13 (19·4%) cases and 8 (11·9%) controls (p = 0·34). When we analyzed the valid retinograhies, we did not find significant differences between cases (AVR: 0·8487 ± 0·0822) and controls (AVR: 0·8474 ± 0·0835; p = 0.93).

Table F in S1 File shows the results of the multiple regression analysis for all the population that accepted to participate in the study, including matched and unmatched individuals. Classic cardiovascular risk factors (i.e: age, male sex, higher BMI, diabetes) correlated significantly with 24-h maximal systolic BP values, while diagnosis of COPD did not.

## Discussion

In this study we have investigated a possible mechanism for the link between COPD and CVD: hypertension induced by sympathetic overactivity. However, we have not found differences in BP or catecholamines values between patients with COPD and matched controls.

CVD are a major contributor to mortality in COPD. Actually, COPD might be an independent risk factor for CVD[2–4,24–26]. However, some studies have failed to confirm the association between COPD and CVD, and some authors have suggested that this association might be explained by the presence of shared risk factors [5]. One strong point of our investigation is that study subjects were matched regarding age, sex and smoking history, variables that influence differences in cardiovascular mortality. Also, classical cardiovascular risk factors were similarly distributed between both groups, including socioeconomic aspects and physical activity level.

Proposed mechanisms for the association between CVD and COPD include systemic inflammation, hypoxia, oxidative stress, connective tissue alteration, augmented platelet activation and impaired endothelial function [6]. Sympathetic overactivity has also been proposed as a relevant mechanism [27], and some studies reported increased plasma norepinephrine concentrations in COPD patients [28,29].

If sympathetic overactivity would be present in COPD, possible consequences would include endothelial dysfunction, arterial stiffness, left ventricular hypertrophy (LVH), arrhythmias and hypertension [27]. Few studies have evaluated differences in BP between COPD patients and controls. Anderson et al found that 24-h, daytime and nighttime systolic BP values were higher in 93 normoxemic COPD patients than in 34 control subjects [12]. However, both study groups were not well matched concerning age, history of previous hypertension and smoking. Most importantly, 55·9% of control subjects were never-smokers [12]. Aidar et al performed arterial BP monitoring and polysomnography in 13 COPD patients and 14 controls and found higher blood pressure values, and higher CT90 in the COPD group. However, both groups were not matched regarding smoking history [13]. The present study has not found differences in BP values, or urine catecholamine levels between COPD patients and matched controls. Our findings suggest, consequently, that hypertension due to sympathetic overactivity is not a relevant mechanism explaining the association between COPD and CVD.

We must recognize the fact that catecholamines measurement might be relatively insensitive to detect sympathetic overactivity. Monoamines excretion is susceptible to daily variations within subjects [18]. Also, there can be a wide variation in the excretion patterns of catecholamines between individuals [18]. This raises the possibility that our study might be underpowered to detect differences between cases and controls. Several studies have studied sympathetic activity in patients with respiratory diseases by means of measuring muscle sympathetic nerve activity, as recorded by microneurography [9,30–31]. Heindl et al found increased muscle sympathetic nerve activity in 11 patients with chronic hypoxia due to COPD and pulmonary fibrosis, compared with control subjects. Oxygen administration decreased sympathetic activity in patients with hypoxia, linking sympathetic overactivity to chemoreflex activation by hypoxemia [9]. Ashley et al found an increase in central muscle vasoconstrictor drive in 18 patients with COPD [30]. However, none of these patients were hypertensive, in contrast with a previous similar study carried out in patients with OSA [30]. In another study from the same group, the systolic, diastolic and mean BP values of 15 COPD patients were similar to those of 10 healthy controls with a mean age of 50 years [31]. The authors hypothesized that the persistent hypoxia (and, in some cases, hypercapnia) seen in COPD patients might cause a vasodilator effect that might counteract the effect of sympathoexcitation and temper any tendency towards neurogenic hypertension [30].

A secondary objective of our study was to search for differences suggestive of hypertensive organ damage between cases and controls. Anderson et al previously found LVH in 30·1% of normoxemic COPD patients [12]. Echocardiographies in our study were similar between cases and controls. There was, however, a tendency towards lower LV ejection fraction in the COPD group. Possible mechanisms implicated in alterations of LV function in COPD are complex, and not necessarily related to systemic or coronary vascular damage. Pulmonary hyperinflation and intrinsic positive end-expiratory pressure might reduce intrathoracic blood volume and impair LV function due to a decrease in LV preload [32].

Hypertensive retinopathy has been regarded as a marker of systemic vascular disease [33]. Retinal-arteriolar narrowing could be an early marker of overt hypertension, and might predict the subsequent development of hypertension in subjects previously classified as normotensive [33]. Therefore, retinography could plausibly be used as a sensitive marker of early organ damage due to high BP values. We found similar values in the retinal AVR between both study groups. This is in accordance with the other findings of the study, and indicates that hypertension prevalence is not higher in COPD.

Several limitations of our study must be considered. The most important are its single-center nature and relatively small sample size, which limit the generalisability of the results. The control subjects had a lesser degree of tobacco exposure than the COPD patients, but this could have plausibly biased the results towards higher BP values in the COPD group. The absence of differences between both groups cannot be attributed to this fact. We must emphasize the fact that the control group cannot be considered healthy, given the history of smoking. Our intention when we decided to study subjects with a history of smoking was to avoid the confusing factor of tobacco. However, it is plausible that the inclusion of an additional control group of age- and sex-matched subjects who did not smoke might had revealed differences with respect to COPD cases. The heterogeneity in disease severity between the COPD cases is also a limitation. There were relatively few cases with mild COPD and few patients with very severe airway obstruction (i.e: FEV_1_ < 30%), and this is a relevant limitation. However, CVD incidence is high in all stages of COPD, and death due to CVD is frequent in both mild and moderate COPD patients [34]. Therefore, we think that this limitation does not invalidate our conclusions. When programming the 24 h-BP monitor, we did not take into account each subject’s sleep habits, but used instead a fixed nighttime and daytime period for all participants, and this might have introduced some degree of uncertainty in our results. We did not perform polysomnographies and, consequently, OSA could not be ruled out. OSA is associated with an increased risk of hypertension.[8] If there would have been more OSA cases in the control group, this would have reduced the differences in BP between both study groups. However, there were no differences in the values of the Epworth sleepiness scale. Also, nocturnal oximetry was performed in a representative sample of COPD cases and controls, and a “saw-tooth” pattern (a finding suggestive of OSAS) was found with similar frequency in both groups. Thus, significant bias in this respect is not probable.

In conclusion, our study suggests that BP and catecholamines values are similar between COPD patients and control subjects with similar prevalence of classical cardiovascular risk factors. These results suggest that hypertension induced by sympathetic overactivity is not a significant mechanism explaining the link between COPD and CVD. The study does not provide additional evidence for the hypothesis of COPD as an independent risk factor for CVD, beyond other recognized cardiovascular risk factors.

## Supporting Information

S1 File Supplemental Tables A to F. (DOC)
