# Supplementary material for: Blood Pressure Profile and Hypertensive Organ Damage in COPD Patients and Matched Controls. The RETAPOC Study
Source: PLoS One. 2016 Jun 30;11(6):e0157932. doi: 10.1371/journal.pone.0157932 (PMC4928916; doi:10.1371/journal.pone.0157932)
Supplement: S1 File — (DOC) [file pone.0157932.s001.doc]

**SUPPLEMENTARY MATERIAL**

**Table A: Differences between COPD patients matched with controls and those lost to matching process**

| **Variable** | **Matched cases** | **Subjects lost to matching** | **P** |
| --- | --- | --- | --- |
| Age, yr | 59·4 ± 5·9 | 64·1 ± 3·8 | < 0·0001 |
| Males, % | 77·6% | 100% | 0·0003 |
| Charlson index | 2·0 ± 1·1 | 1·5 ± 0·8 | 0·06 |
| Active smokers, % | 50·7% | 60.0% | 0·38 |
| Pack-years | 52·7 ± 24·4 | 60·8 ± 32·3 | 0·26 |
| BMI, Kg/m^2^ | 27·7 ± 4·5 | 29·1 ± 5·0 | 0·11 |
| Abdominal perimeter, cm | 100·4 ±12·3 | 104·4 ± 12·8 | 0·07 |
| FEV_1_ % | 58·4 ± 20·7 | 61·0 ± 20·5 | 0·44 |
| FVC % | 82·3 ± 16·8 | 83·5 ±16·1 | 0·77 |
| FEV1/FVC % | 51·3 ± 12·3 | 51·9 ±14·2 | 0·56 |
| 24 h mean SBP, mmHg | 124·8 ±12·6 | 126·9 ± 11·0 | 0·19 |
| 24 h maximal SBP, mmHg | 155·3 ±17·2 | 160·0 ± 15·5 | 0·04 |

BMI: body mass index; SBP: systolic blood pressure

**Table B: results of the ambulatory blood pressure monitoring for subjects without previous treatment for hypertension.**

| **Variable** | **Cases (n = 38)**  **COPD** | **Controls (n = 42)**  **Non-COPD smokers** | **P** |
| --- | --- | --- | --- |
| 24 h mean SBP, mmHg | 122·9 ± 9·.9 | 121·7 ± 12·1 | 0·62 |
| 24 h maximal SBP, mmHg | 152·3 ± 13·2 | 148·5 ± 16·3 | 0·26 |
| 24 h mean DBP, mmHg | 75·4 ± 7·2 | 74·8 ± 6·8 | 0·73 |
| 24 h maximal DBP, mmHg | 99·3 ± 10·5 | 99·6 ± 9·7 | 0·24 |
| Daytime mean SBP, mmHg | 126·1 ± 11·1 | 125·8 ± 12·1 | 0·91 |
| Daytime maximal SBP, mmHg | 152·2 ± 13·3 | 148·4 ± 16·4 | 0·26 |
| Daytime mean DBP, mmHg | 78·8 ± 8·2 | 78·4 ± 7·3 | 0·82 |
| Daytime maximal DBP, mmHg | 99·1 ± 10·6 | 96·3 ± 9·9 | 0·23 |
| Nighttime mean SBP, mmHg | 116·8 ± 11·6 | 115·1 ± 2·0 | 0·53 |
| Nighttime maximal SBP, mmHg | 137·3 ± 12·0 | 134·3 ± 17·0 | 0·36 |
| Nighttime mean DBP, mmHg | 69·4 ± 7·5 | 69·3 ± 7·1 | 0·92 |
| Nighttime maximal DBP, mmHg | 86·1 ± 10·3 | 84·3 ± 10·6 | 0·46 |
| Non dipper, n, % | 20 (52·6%) | 25 (59·5%) | 0·59 |
| Dipper, n, % | 14 (36·8%) | 14 (33·3%) |  |
| Extreme dipper, n,% | 0 | 1 (2·3%) |  |
| Riser, n,% | 4 (10·5%) | 2 (4·7%) |  |

SBP: systolic blood pressure; DBP: diastolic blood pressure.

**Table C: Prevalence of occult hypertension in subjects without previous treatment for hypertension, according to different criteria.**

| **Variable** | **Cases (n = 38)**  **COPD** | **Controls (n = 42)**  **Non-COPD smokers** | **P** |
| --- | --- | --- | --- |
| 24 h mean BP > 130/80 mm Hg | 7 (18·4%) | 6 (14·2%) | 0·83 |
| Daytime mean BP > 135/85 mmHg | 13 (34·2%) | 15 (35·7%) | 0·92 |
| Nighttime mean BP > 120/75 mmHg | 17 (44·7%) | 13 (30·9%) | 0·29 |
| Any of the 3 criteria | 19 (50%) | 17 (40·4%) | 0·52 |

BP: blood pressure.

**Table D: differences between subjects who had oximetry performed and those with no oximetry results**

|  | **Cases (n= 67) COPD** | | | **Controls (n = 67) smokers, non-COPD** | | |
| --- | --- | --- | --- | --- | --- | --- |
| **Variable** | **Oximetry performed (n = 40)** | **Oximetry not performed (n= 27)** | **P** | **Oximetry performed (n =32)** | **Oximetry not performed (n = 35)** | **p** |
| Age, yr | 59·2 ± 5·4 | 59·6 ± 6·7 | 0·77 | 57·6 ± 7·3 | 58·7 ± 6·6 | 0·53 |
| Males, n, % | 29 (72·5%) | 23 (85·2%) | 0·35 | 26 (81·2%) | 26 (74·3%) | 0·69 |
| Modified Charlson index | 0·89 ± 1·14 | 1·11 ± 1·18 | 0·46 | 0·53 ± 0·67 | 0·88 ± 1·07 | 0·18 |
| Pack-years | 52·1 ± 25·6 | 53·5 ± 23·0 | 0·82 | 42·0 ± 31·8 | 45·3 ± 21·8 | 0·64 |
| BMI, kg/m^2^ | 27·4 ± 4·3 | 28·1 ± 4·9 | 0·54 | 29·7 ± 4·8 | 27·7 ± 4·3 | 0·65 |
| FEV_1_ % | 59·6 ± 20·8 | 56·7 ± 20·6 | 0·57 | 96·2 ± 16·7 | 97·0 ± 15·1 | 0·84 |
| FVC % | 86·4 ± 14·0 | 76·5 ± 19·9 | 0·01 | 95·8 ± 13·5 | 93·2 ± 13·8 | 0·43 |
| FEV1/FVC % | 49·8 ± 12·9 | 53·4 ± 11·1 | 0·25 | 77·2 ± 4·5 | 76·9 ± 4·1 | 0·77 |
| 24 h mean SBP, mmHg | 124·8 ± 14·3 | 124·9 ± 9·9 | 0·47 | 123·9 ± 12·5 | 125·3 ± 12·9 | 0·98 |
| 24 h maximal SBP, mmHg | 154·9 ± 18·9 | 155·8 ± 14·7 | 0·52 | 154·4 ± 18·9 | 155·2 ± 18·1 | 0·87 |

BMI: body mass index, SBP: systolic blood pressure.

**Table E: correlation between catecholamines and 24 h blood pressure**

| Variable | Correlation coefficient (95% CI) | p |
| --- | --- | --- |
| 24 h- maximal SBP | | |
| Epinephrine | 0·15 (-0·02, 0·32) | 0·08 |
| Norepinephrine | 0·20 (0·02, 0·36) | 0·02 |
| Metanephrine | 0·02 (-0·15, 0·20) | 0·77 |
| Normetanephrine | 0·03 (-0·14, 0·21) | 0·72 |
| 24 h-mean SPB | | |
| Epinephrine | 0·14 (-0·04, 0·31) | 0·12 |
| Norepinephrine | 0·18 (0·01, 0·35) | 0·04 |
| Metanephrine | -0·01 (-0·18, 0·17) | 0·96 |
| Normetanephrine | -0·02 (-0·20, 0·15) | 0.79 |
| 24 h-maximal DBP | | |
| Epinephrine | 0·05 (-0·12, 0·23) | 0·54 |
| Norepinephrine | 0·03 (-0·14, 0·21) | 0·73 |
| Metanephrine | -0·01 (-0·18, 0·18) | 0·99 |
| Normetanephrine | 0·01 (-0·17, 0·18) | 0.97 |
| 24 h-mean DBP | | |
| Epinephrine | 0·16 (-0·02, 0·33) | 0·07 |
| Norepinephrine | 0·16 (-0·02, 0·32) | 0·08 |
| Metanephrine | 0·38 (-0·10, 0·26) | 0·38 |
| Normetanephrine | 0·03 (-0·14, 0·22) | 0·69 |

SBP: systolic blood pressure; DBP: diastolic blood pressure.

**Table F: Multiple regression analysis results using 24 maximal SBP as dependent variable, for all the population that accepted to participate in the study, including matched and unmatched individuals**

| **Variable** | **R** | **P** |
| --- | --- | --- |
| Age | 0·18 | 0·008 |
| Male sex | 0·13 | 0·053 |
| BMI | 0·24 | 0·0004 |
| Diabetes | 0·17 | 0·014 |
| COPD diagnosis | 0·04 | 0·54 |

SBP: systolic blood pressure; BMI: body mass index.

The covariates included in the analysis were COPD diagnosis and the main classical cardiovascular risk factors: age, male sex, body mass index –as an indicator of obesity-, diabetes, alcohol intake and dyslipidemia. The alcohol intake and dyslipidemia variables were not retained in the final model, while the p-value for “male sex” variable approached statistical significance. The variance inflation factor values were between 1.083 and 1.125, ruling out multicollinearity.
